# Reducing the burden of preventable deaths from sepsis in Canada: A need for a national sepsis action plan

**DOI:** 10.1177/08404704241240956

**Published:** 2024-04-10

**Authors:** Fatima Sheikh, Victoria Chechulina, Gary Garber, Kathryn Hendrick, Niranjan Kissoon, Laurie Proulx, Kristine Russell, Alison E. Fox-Robichaud, Lisa Schwartz, Kali A. Barrett

**Affiliations:** 1 62703 McMaster University, Hamilton, Ontario, Canada.; 2 6221 Western University, London, Ontario, Canada.; 3 33497 Canadian Medical Protective Association, Ottawa, Ontario, Canada.; 4 6363 University of Ottawa, Ottawa, Ontario, Canada.; 5 Sepsis Canada Patient Advisory Council, Hamilton, Ontario, Canada.; 6 37210 BC Children’s Hospital, Vancouver, British Columbia, Canada.; 7 8166 University of British Columbia, Vancouver, British Columbia, Canada.; 8 Canadian Arthritis Patient Alliance, Ottawa, Ontario, Canada.; 9 2129 University of Calgary, Calgary, Alberta, Canada.; 10 7938 University of Toronto, Toronto, Ontario, Canada.

## Abstract

Sepsis is a global health threat with significant morbidity and mortality. Despite clinical practice guidelines and developed health systems, sepsis is often unrecognized or misdiagnosed, leading to preventable harm. In Canada, sepsis is responsible for 1 in 20 deaths and is a significant driver of health system costs. Despite being a signatory to the World Health Organization’s Resolution WHA 70.7, adopted in 2017, Canada has not lived up to its commitment. Many existing sepsis policies were developed in response to a specific tragedy, and there is no national sepsis action plan. In this article, we describe the burden of sepsis, provide examples of existing, context-specific, reactionary sepsis policies, and urge a coordinated, proactive Canadian sepsis action plan to reduce the burden of sepsis.

## Sepsis is a public health emergency in Canada

Sepsis is a clinical syndrome characterized by organ dysfunction in the setting of an infection. In 2017, it affected 48.9 million people and killed 11 million people worldwide.^1,2^ Because sepsis syndrome can develop in response to different types of infection, it is often overlooked. For example, although rarely reported or acknowledged as such, SARS-CoV-2 infection can cause sepsis.^3^ Even before the COVID-19 pandemic, sepsis represented a significant economic burden for the Canadian healthcare system.

Despite advances in medicine and public health over the past decades, sepsis continues to cause harm and consume healthcare dollars even after the first hospital encounter.^4-6^ A recent study exploring the prevalence of sepsis among hospitalizations in Ontario found that approximately 1 in 6 hospitalizations involve an episode of sepsis and 1 in 20 hospitalizations involve severe sepsis.^7^ Those with severe sepsis had a longer hospital length of stay, expensive hospitalizations (mean cost of $23,530 CDN, 95% CI $22,974 - $24,019), and an increased risk of readmission for up to 5 years. In Ontario, annual costs due to sepsis were estimated to be more than 1 billion dollars.^7^

Amid an Emergency Department (ED) crisis,^8,9^ the importance of public health and policy measures to support early detection of sepsis in EDs cannot be understated. To date, clinical studies on sepsis have primarily investigated its pathogenesis, established its diagnostic criteria, and determined how to manage sepsis in the hospital setting.^10^ However, in 80% of cases, the onset of sepsis occurs outside of healthcare settings.^11^ This highlights the need for efforts to reduce infection rates and improve early detection in pre-clinical settings and hospital EDs.^11,12^

Prior to the COVID-19 outbreak, scientists and public health officials warned that Canada was not prepared for the next pandemic.^13^ COVID-19 subsequently demonstrated significant vulnerabilities in the Canadian health system. Canada’s mortality rate from COVID-19 exceeded the global average, with the majority of deaths occurring among those from systematically marginalized communities and individuals living in Long-Term Care (LTC) homes.^14^ Policy-makers are now beginning to reflect on the vulnerabilities in the health system and working to identify areas for improvement. In the same way that Canada was not prepared for the COVID-19 pandemic, Canada does not have a national sepsis action plan to effectively reduce the human and health economic burden of sepsis.

In 2017, the World Health Assembly (WHA) passed a resolution that urges member states, including Canada, to take specific actions to reduce the burden of sepsis.^15^ A key pillar of resolution WHA70.7, “Improving the prevention, diagnosis and clinical management of sepsis”, includes defining and implementing standards and establishing guidelines to facilitate prevention, early diagnosis, and appropriate and timely clinical management of sepsis. Although the implementation of national sepsis policies, guidelines and clinical standards has been associated with a reduction in sepsis mortality in other countries, including Australia,^16^ Ireland,^17^ the United Kingdom,^18^ and the United States^19,20^ there is no similar national sepsis strategy in Canada.

Sepsis prevention policies exist in Canada, but many are developed in response to a tragedy and remedy specific policy gaps. Below, we present three examples of sepsis policies that demonstrate this kind of reactionary policy making. The first refers to food safety standards, the second to LTC standards, and the third is an example of a comprehensive sepsis prevention policy for hospitals. They are drawn from both the United States and Canada and were implemented by both national and regional levels of government. We are not critical of the policies themselves, but they demonstrate how extant sepsis policy develops from tragedy, a pattern that needs to change.

## Food safety policies to prevent sepsis in the Unites States and Canada following sepsis-related deaths caused by food-borne pathogens

In 1992, an outbreak of *Escherichia coli (E. coli)* O157:H7 originating from contaminated hamburgers sold at Jack-in-the-Box resulted in more than 600 people becoming ill with sepsis, and the tragic death of four children who developed severe sepsis and multi-organ failure.^21^ This outbreak received significant media coverage and public outrage. There was a swift response from lawmakers to enact policies to improve the safety of the food supply. *E*.*coli* O157:H7 was officially listed as a food adulterant in raw ground beef, a zero-tolerance policy for fecal contamination of beef carcasses was passed, and national enforcement of the Hazard Analysis and Critical Control Point system (HACCP), a quality control method to improve food safety, was implemented.^21^

A subsequent *E*. *coli* outbreak in 2007 infected more than 100 individuals in the United States was found to originate from Canadian beef farmed in Alberta.^22^ The US Food Safety and Inspection Service (FSIS) investigated the outbreak and determined that the food safety system in Canada had failed. They identified that many Canadian food supply establishments were not in compliance with the HAACP system and that when bacterial contamination was identified, the Canadian Food Inspection Agency (CFIA) had failed to follow up properly on these findings. Following this outbreak, compliance with the HACCP system was enforced in Canada and policies were changed so that all beef trimmings destined for the United States were tested for contamination prior to export.

The food safety system, like the health system, exists to protect the public and promote health and well-being. Both systems require comprehensive and effective policies and regulations. The Jack-in-the-Box outbreak “changed the history of food safety” as it revealed serious system-level failures and led to global changes in food safety regulation.^21^

## New LTC standards to prevent sepsis following deaths due to COVID-19

The COVID-19 pandemic was devastating for older adults living in LTC homes in Canada.^23^ In Ontario, despite only representing .5% of the total population, 54% of all COVID-19 deaths between March 2020 and March 2021 were among LTC residents.^24^ The vulnerabilities of the Canadian LTC system were known prior to the COVID-19 pandemic.^13^ There were many underlying contributing factors to these deaths, including a public health failure early in the pandemic to consider the risk of community spread of the virus and the subsequent spread to LTC residents, an insufficient supply of personal protective equipment for healthcare workers and residents, resident overcrowding, staff shortages, system underfunding, and a lack of system integration and coordination.^13,24,25^ In some situations, the Canadian Armed Forces had to be recruited to help provide care to LTC residents.

Following the devastation in Ontario LTC homes, the Ontario COVID-19 Long-Term Care Commission was established. Recommendations in their April 2021 report included a call to adhere to the precautionary principle, having a pandemic plan and stockpile of resources for the future, improved infection control practices, better system integration, efforts to build and maintain a sustainable workforce, and increased LTC system funding.^24,26^

The COVID-19 disaster in LTC homes was also an impetus for the development of long-overdue national standards for LTC facilities. The Health Standards Organization developed a National Health Standard for Long-Term Care Services with the aim of “promoting good governance; upholding resident-centred care and enabling a meaningful quality of life for residents; ensuring high-quality and safe care; fostering a healthy and competent workforce; and promoting a culture of quality improvement and learning across long-term care.”^27^

The Canadian Standards Association (CSA) simultaneously developed national standards for infection prevention and control in Canadian LTC facilities, providing “guidance on safe operating practices, design, and IPAC in LTC homes while incorporating a person-centred approach. The standard takes into consideration what is required during both normal, day-to-day circumstances and catastrophic events (e.g., outbreaks, epidemics, pandemics, fires, earthquakes, and loss of power)”.^28^

The COVID-19 pandemic resulted in a reprehensible number of deaths in Canadian LTC homes. These long-overdue national standards will hopefully ensure that conditions in LTC homes promote health and well-being and prevent future deaths from sepsis by directly addressing the policy failures that led to the spread of SARS-CoV-2 within LTC homes, including the breakdown of staffing models that left vulnerable residents at greater risk of infection and deterioration.

## State-level sepsis policies following the death of a child due to sepsis: Rory’s regulations

In April 2012, Rory Staunton, a 12-year-old boy from New York, died of sepsis. Just a few days earlier, he had cut his arm during gym class, fell ill, and despite multiple visits to his paediatrician and the emergency department, his signs and symptoms of sepsis went unrecognized. Just three days later, Rory, a previously healthy child, passed away from sepsis.^29^ This story received significant media coverage, including stories in the *New York Times*,^30^ *USA Today*,^31^ and a feature in *People* magazine.^32^

Following his death, Rory’s parents, Ciaran and Orlaith Staunton, established the Rory Staunton Foundation. His family, like many, had never heard of sepsis, but committed to ensuring that no other child or family endured the loss they experienced. Now renamed End Sepsis, this foundation advocates on behalf of families who “demand better infection education and hospital safety measures to ensure there are no more needless deaths from sepsis, a preventable and treatable disease.”^33^

The Stauntons, including Rory Staunton, were politically well-connected. Just 2 weeks before Rory’s death, Rory and his father were at the White House where he had met President Obama and Vice President Biden. Effective lobbying, specifically through hierarchical ties with political leaders and an identifiable target of influence, helped ensure that the Stauntons’ concerns were reflected in policy and the decision to mandate sepsis protocols.^34^ Under the Stauntons’ leadership, The Rory Staunton Foundation helped achieve many key milestones, including the adoption of Rory’s regulations in January 2013, the first United States Senate hearing on sepsis in September 2013, and the inclusion of sepsis on the CDC web site.

New York State Governor Andrew Cuomo passed “Rory’s Regulations” in 2013. These regulations require all New York hospitals to develop protocols for the early identification and treatment of sepsis. Specifically, according to Title 10 of the New York State Codes, Rules, and Regulations (Sections 405.2 and 405.4) acute care hospitals in New York are required to (1) adopt and implement evidence-based protocols for early recognition and treatment of sepsis; (2) train staff to appropriately implement sepsis protocols; and (3) collect and report quality measures related to the recognition and treatment of sepsis for internal quality measurements and reporting to the New York Department of Health.

Ultimately, the unexpected and preventable death of a child and the associated media coverage of the tragedy were arguably the leading factors that contributed to the passage of Rory’s regulations. Following the success in New York,^19,35^ 17 other states, including Illinois in 2016 and New Jersey in 2018, have implemented strategies to reduce the burden of sepsis.^36,37^ There are no similar national, provincial, or territorial policies in Canada. Additionally, there are no standards specific to sepsis in Canada; hospital standards developed by Accreditation Canada only discuss infection prevention and control more generally.^38,39^

## Canada needs proactive sepsis policy

We believe that a Canadian sepsis action plan would help prevent new cases of sepsis, save lives, and reduce the burden of sepsis on the Canadian health system. In each of the three examples above, sepsis policies were developed in response to deaths from sepsis. We envision a future where there are similar but more comprehensive and coordinated health policies to reduce the burden of sepsis in the country.

In British Columbia a provincial quality improvement initiative designed to reduce sepsis occurrences prevented an estimated 981 sepsis cases and 172 deaths and saved an estimated 50.6 million dollars in 2018 alone. It is estimated that $1.00 invested in this program, saved $112.50 of healthcare costs.^40^ The economic burden of sepsis extends well beyond the hospital stay, including the impact on families, and further strengthens the argument for policy efforts to reduce the occurrence of sepsis.^41^ National standards, like those implemented in LTC settings, are an opportunity to implement the British Columbia model of care within Canada’s federally funded and provincially administered healthcare system. There is both a role and a responsibility for the Canadian federal government to support efforts to reduce the burden of sepsis and act upon the WHA resolution on sepsis.

Cognizant of the slow progress globally, on September 12, 2023, the Global Sepsis Alliance and Sepsis Stiftung released the Berlin Declaration on Sepsis, reiterating an urgent call for the enforcement of the WHA 70.7 resolution on sepsis, signed by 76 other sepsis organizations.^42^ The Berlin Declaration calls on UN member states to meet the following five pillars:

(1) to include sepsis prevention, diagnosis, and treatment in community and healthcare settings;
(2) to implement standards for diagnosing and managing sepsis in health emergencies;
(3) to increase public awareness;
(4) to develop training standards for all health professionals to recognize, and appropriately treat sepsis early; and
(5) to promote innovative research to address sepsis across the lifespan.

Included in the Berlin Declaration is a call to the director general of the World Health Organization, key stakeholders in global health, and G7 and G20 leaders to increase efforts to detect, diagnose, and manage sepsis early and appropriately.

In alignment with pillar 5, in 2019 the Canadian Institutes of Health Research funded Sepsis Canada, a national interdisciplinary research network to study sepsis across the biomedical, clinical, health services, and population health pillars. This research network has made progress in developing much-needed research infrastructure. The network has funded teams of scientists and engaged patient and family partners across the country whose work will provide important contributions to the field of sepsis.

Canada can save lives by turning its attention to the remaining four pillars in a coordinated and proactive fashion. We are a multidisciplinary team of people with lived experience of sepsis, researchers, and health professionals who are eager to support national, provincial, and territorial regulatory and health system leaders to proactively develop comprehensive and evidence-based standards for the prevention, identification, and treatment of sepsis in Canada, in alignment with pillars 1, 2, 3 and 4.

This type of policy development cannot start without an understanding of what already exists. The current landscape of Canadian sepsis policies is not well described. We are leading a scoping review to identify and describe existing sepsis policies, clinical practice guidelines, and health professional training standards in Canada, and, more importantly, to identify crucial policy gaps that need attention.^43^

We cannot wait for the next disaster to strike to begin this work. We ask that Canadian provincial, territorial, and federal ministers and health system leaders recognize the impact sepsis has on our health system and support and promote efforts to develop an evidence-based national sepsis action plan that seeks to prevent new cases of sepsis and improve recognition and treatment for those with sepsis during both their acute illness and their recovery.

## Conclusion

Canada, as a signatory to the WHA resolution, must fulfil its obligation to reduce the burden of sepsis within our borders. Although existing policies that seek to prevent sepsis, such as the food safety policies and LTC standards described above, are reactionary and address the specific failure points of that specific system, they are examples of what can be done to mitigate the burden of sepsis and an opportunity to build on. Despite the successful implementation of national sepsis policies and programs around the world, and the success of state-level policies in New York, there are no similar policies in Canada. As Canada grapples with health system challenges, and an ageing population at increased risk of sepsis,^44^ a coordinated response is needed to reduce the burden of sepsis. We call on Canadian healthcare decision-makers to work with us to develop a national sepsis action plan and ensure compliance with the WHA resolution and the Berlin Declaration.
